# *Mycobacterium tuberculosis* Is a Natural Ornithine Aminotransferase (*rocD*) Mutant and Depends on Rv2323c for Growth on Arginine

**DOI:** 10.1371/journal.pone.0136914

**Published:** 2015-09-14

**Authors:** Annegret Hampel, Claudia Huber, Robert Geffers, Marina Spona-Friedl, Wolfgang Eisenreich, Franz-Christoph Bange

**Affiliations:** 1 Department of Medical Microbiology and Hospital Epidemiology, Medical School Hannover, 30625 Hanover, Germany; 2 Lehrstuhl für Biochemie, Technische Universität München, Garching, Germany; 3 Research Group Genome Analytics, Helmholtz Center for Infection Research, 38124 Braunschweig, Germany; Institut de Pharmacologie et de Biologie Structurale, FRANCE

## Abstract

*Mycobacterium tuberculosis* (Mtb) possesses a genetic repertoire for metabolic pathways, which are specific and fit to its intracellular life style. Under *in vitro* conditions, Mtb is known to use arginine as a nitrogen source, but the metabolic pathways for arginine utilization have not been identified. Here we show that, in the presence of arginine, Mtb upregulates a gene cluster which includes an ornithine aminotransferase (*rocD*) and Rv2323c, a gene of unknown function. Isotopologue analysis by using ^13^C- or ^15^N-arginine revealed that in Mtb arginine is not only used as nitrogen source but also as carbon source for the formation of amino acids, in particular of proline. Surprisingly, *rocD*, which is widespread in other bacteria and is part of the classical arginase pathway turned out to be naturally deleted in Mtb, but not in non-tuberculous mycobacteria. Mtb lacking Rv2323c showed a growth defect on arginine, did not produce proline from arginine, and incorporated less nitrogen derived from arginine in its core nitrogen metabolism. We conclude that the highly induced pathway for arginine utilization in Mtb differs from that of other bacteria including non-tuberculous mycobacteria, probably reflecting a specific metabolic feature of intracellular Mtb.

## Introduction

*Mycobacterium tuberculosis* (Mtb) is responsible for 9 million people with active tuberculosis annually, resulting in 1.5 million death cases per year worldwide [1]. Inside the host, the pathogen faces nitrosative, oxidative, and acidic stress as well as a limited supply of nutrients [2–4]. Mice and murine cell lines produce a strong nitrosative burst, which is an essential defense mechanism for controlling Mtb infection [5,6]. In infected human macrophages, nitric oxide production is demonstrated, yet NO levels are not bactericidal for the pathogen [7–9]. Instead, it has been suggested that nitrate, which stems from host-derived NO, is metabolized by the pathogen itself, and enhances the survival of Mtb inside macrophages [10–12]. Arginine is the substrate for the host’s NO production during nitrosative burst [13,14] and its uptake is increased in activated macrophages [15,16]. In contrast to a leucine auxotrophic mutant of Mtb that is cleared from infected mice, an arginine auxotrophic strain of Mtb is attenuated, yet still able to grow in mice at later stages of infection [17,18]. This indicates that Mtb is able to access arginine, which might be used by the pathogen as nitrogen or carbon source.

In the central nitrogen metabolism, ammonia is assimilated via the glutamate dehydrogenase (GDH) pathway producing glutamate from α-ketoglutarate. Alternatively, the glutamine synthetase / glutamate synthase (GS/GOGAT) pathway transfers ammonia to glutamate producing glutamine, which subsequently can be converted into two molecules of glutamate in the presence of α-ketoglutarate [19]. Glutamate and glutamine are the key metabolites in the central nitrogen metabolism; both serve as endogenous nitrogen acceptor as well as nitrogen donor. Recently, asparagine has been suggested to provide nitrogen for Mtb during infection in mice [20]. Yet, Mtb can utilize several amino acids as a source of nitrogen *in vitro*, including arginine [21]. At present, the molecular mechanisms for arginine utilization in Mtb are not known.

The genome of Mtb includes transporters for arginine uptake, Rv0522 (*gabP*) and the *rocE* homologue [22,23]. For arginine utilization, four major pathways have been described in other bacteria: the arginine deiminase-, arginase-, arginine decarboxylase-, and arginine succinyltransferase pathway [24,25]. Mtb possesses homologues for the arginine deiminase pathway (*arcA*), the arginase pathway (*rocD*, *rocA*), and the arginine decarboxylase pathway (*adi/ speA*) [22]. Previously, we showed in Mtb that *arcA*, encoding for an arginine deiminase, cleaves arginine under anaerobic conditions *in vitro*, resulting in the release of ammonia. However, *arcA* did not mediate nitrogen assimilation from arginine under aerobic growth conditions [21]. At present, the other two pathways have not been studied in Mtb. In *Bacillus subtilis (B*. *subtilis)*, the arginase pathway consists of the *rocDEF* as well as the *rocABC* gene cluster (arginine ornithine catabolism) and allows the conversion of arginine into glutamate, in three steps [26,27]. Arginine is hydrolyzed by an arginase (*rocF)* to ornithine and urea. Ornithine is cleaved to glutamate semialdehyde (GSA) via *rocD* (ornithine aminotransferase). Glutamate semialdehyde (spontaneously cyclizes to form pyrroline-5-carboxylate, P5C) is converted into glutamate by pyrroline-5-carboxylate dehydrogenase, which is encoded by *rocA*. Genes with homology to *rocD* and *rocA* but not to *rocF* and *rocBC* have been found on the chromosome of Mtb [22]. Studies in *Yersinia* showed that, the arginine decarboxylase pathway cleaves arginine into agmatine (Agma) via the arginine decarboxylase, *speA*. Decarboxylation of arginine follows the conversion of agmatine into N-carbamoylputrescine (CP) and putrescine (Put) providing ammonia (*aguA*, *aguB*, *speB*) [28]. The genome of Mtb includes a putative arginine decarboxylase (*adi / speA*) [22].

In this study, we found that in the presence of arginine a distinct gene cluster including *rocDE* and Rv2323c is upregulated, suggesting that the classical arginase pathway metabolizes arginine in Mtb. Surprisingly, the *rocD* gene in Mtb carries a partial deletion, which is specific for the Mtb complex as it is not present in non-tuberculous mycobacteria. Isotopologue analysis starting from ^13^C-labeled arginine as a substrate for Mtb showed that arginine can be converted into ornithine, proline, and glutamate. The role of Rv2323c in arginine utilization of Mtb was demonstrated by an Rv2323c-KO mutant, which was impaired in growth as well as in proline and glutamate formation. These data provide evidence for a novel route in arginine metabolism of mycobacteria involving Rv2323c as the key enzyme.

## Results

### Induction of the *roc* gene cluster during growth of Mtb on arginine

**Fig 1:**
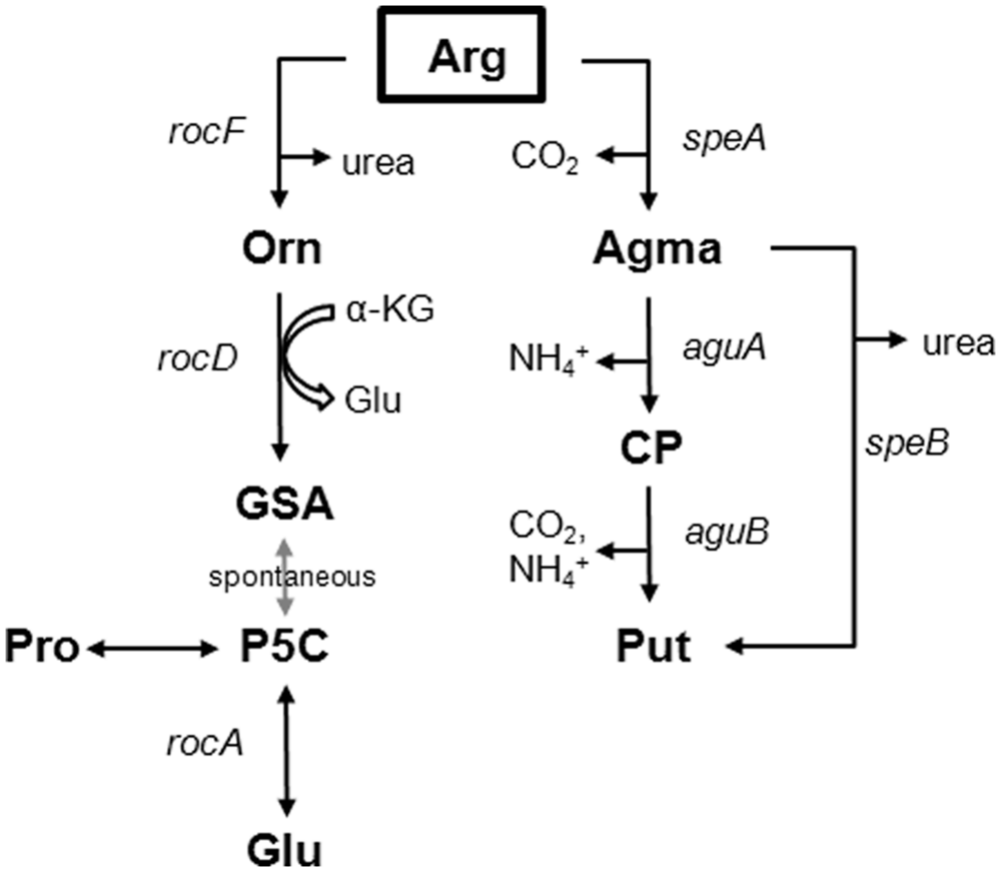
Scheme of the arginase pathway and the arginine decarboxylase pathway. In *Bacillus subtilis*, the first reaction of the arginase pathway, the formation of ornithine (Orn), is catalyzed by arginase (*rocF*). RocD, an ornithine aminotransferase, utilizes ornithine to form glutamate semialdehyde (GSA) or pyrroline-5-carboxylate (P5C). P5C is the substrate of RocA, a pyrroline-5-carboxylate dehydrogenase, which produces glutamate (Glu). In *Yersinia pestis*, an arginine decarboxylase (*speA*) forms agmatine (Agma). Agma is further degraded to N-carbamoylputrescine (CP) and putrescine (Put) by *aguA*, *aguB*, and *speB*. The genome of Mtb includes homologues for the arginase pathway (*rocD*, *rocA*) and the arginine decarboxylase pathway (*speA / adi*).

In other bacteria, at least two pathways, the arginase- and the arginine decarboxylase pathways (Fig 1), are required for arginine metabolism. Arginine induces the expression of several genes involved in arginine uptake and arginine catabolism [26]. To identify genes of the arginine metabolism in Mtb, we performed whole genome expression analysis during growth on arginine. We compared the expression profile of Mtb grown in the presence of arginine with that of Mtb grown in the presence of ammonium and identified 43 genes, which were differentially expressed with an absolute fold change of > 2 and a cut-off significance value of p < 0.05 (S1 Table). Noticeably, the *roc* gene cluster (Rv2319c, *rocE* [Rv2320c], *rocD* [Rv2321c/2322c], Rv2323c) as well as *nirBD* were highly induced in the presence of arginine (more than 20-fold, p < 0.01). The *roc* gene cluster includes the putative arginine permease *rocE*, the ornithine aminotransferase homologue (*rocD*), Rv2319c and Rv2323c, two hypothetical proteins [22], and *nirBD*, a nitrite reductase, has been shown to mediate growth of Mtb on nitrate and nitrite [29].

This induction of *rocDE* during growth on arginine indicates that Mtb possesses the classical arginase pathway for arginine utilization. However, comparing the genome of Mtb with the genomes of other bacteria, Mtb does not possess an arginase homologue (*rocF*), which classically catalyzes the first reaction of the arginase pathway to form ornithine from arginine. Further conversion of ornithine is usually catalyzed by *rocD*, an ornithine aminotransferase, and a *rocD* deficient mutant of *B*. *subtilis* is unable to grow on arginine [26]. Alternatively, arginine can be metabolized via the arginine decarboxylase pathway. Indeed, an arginine decarboxylase deficient *Escherichia coli* strain is impaired for growth on arginine [25,30]. Rv2323c, which was also upregulated in the presence of arginine, shows sequence homology to various hydrolases [31] indicating that Rv2323c could be involved in metabolic processes, in particular in arginine catabolism. Thus, we sought to investigate the importance of *rocD*, *adi* (Rv2531c, an arginine decarboxylase homologue, which in *Yersinia* has been annotated as *speE*), and Rv2323c for the arginine metabolism of Mtb.

### Rv2323c but not *rocD* or *adi* is crucial for growth of Mtb on arginine

**Fig 2:**
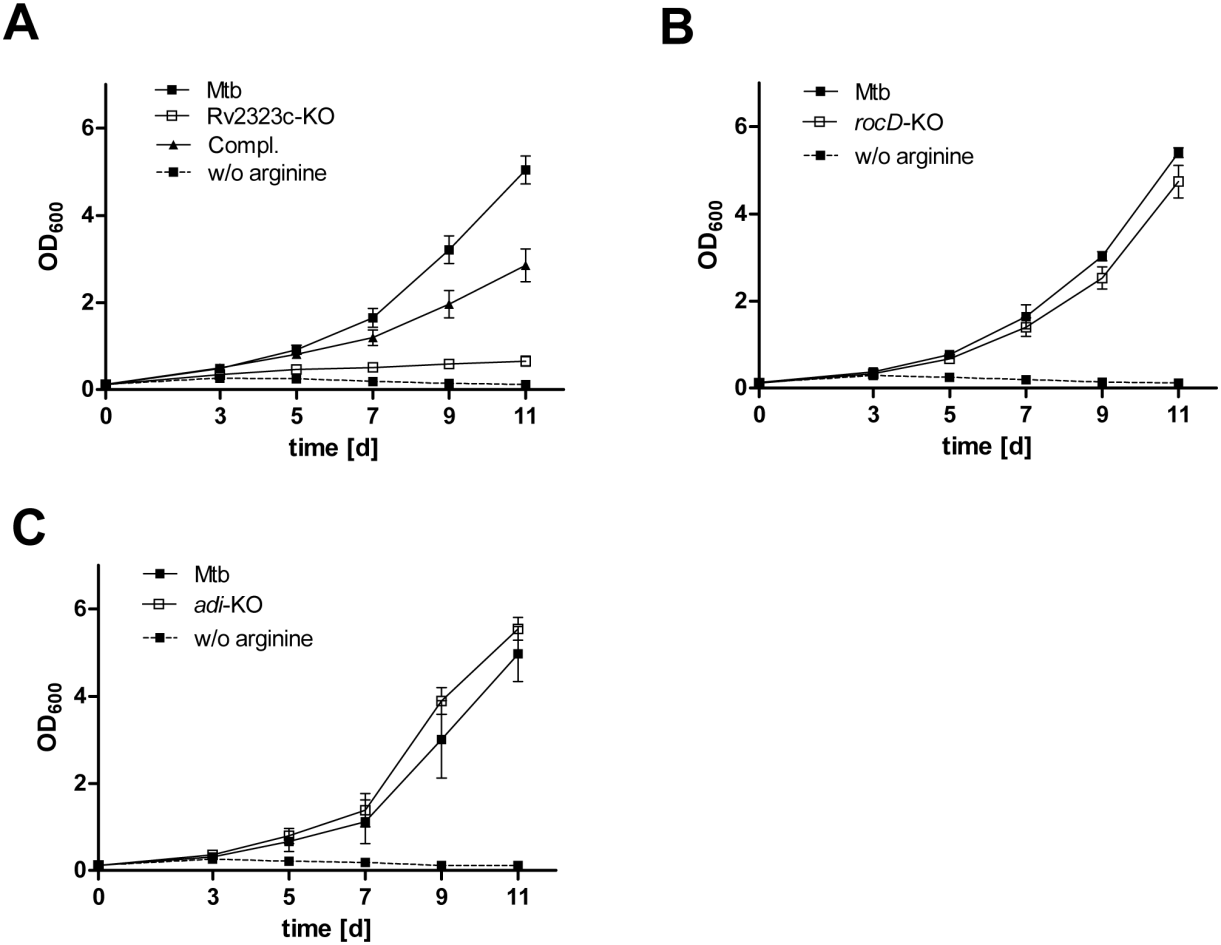
Growth of Mtb knockout strains lacking Rv2323c, *rocD*, or *adi* on arginine as nitrogen source. Mtb wild type (Mtb), Mtb knockout mutants of Rv2323c (Rv2323c-KO) **(A)**, *rocD* (*rocD*-KO) **(B)**, *adi* (*adi*-KO) **(C)**, and the complemented strain for the Rv2323c knockout mutant (Compl.) **(A)** are shown. All strains were cultured for 11 days in minimal medium with glycerol and glucose as carbon sources, and with 5 mM of arginine as a sole nitrogen source (solid line). Mtb wild type was also cultured without arginine as a control (broken line). Growth was analyzed measuring absorbance (OD_600_) at day 3, 5, 7, 9, and 11. Mean values and standard deviations are shown for three independent biological experiments.

To examine the role of *rocD*, *adi*, and Rv2323c for arginine utilization in Mtb, we generated deletion mutants lacking *rocD*, *adi*, or Rv2323c, and investigated the growth of these knockout strains when arginine was provided as sole nitrogen source. Fig 2A shows that growth of an Rv2323c-KO mutant was impaired, indicating a predominant role of Rv2323c for arginine utilization in Mtb. We excluded a general growth defect by showing that the replication of the Rv2323c-KO mutant was unaffected on a different nitrogen source such as glutamate (S1 Fig). In contrast, the *rocD*- and *adi*-KO mutant grew similar to the wild type showing that neither *rocD* nor *adi* is required to metabolize arginine as a sole source of nitrogen (Fig 2B and 2C).

That *rocD* is highly upregulated in the presence of arginine indicates a predominant role of *rocD* in the arginine metabolism of Mtb. Therefore, the unaltered growth of the *rocD*-KO strain was unexpected and raised the question whether *rocD* in Mtb might be non-functional.

### *RocD* in Mtb carries a frameshift mutation

**Fig 3:**
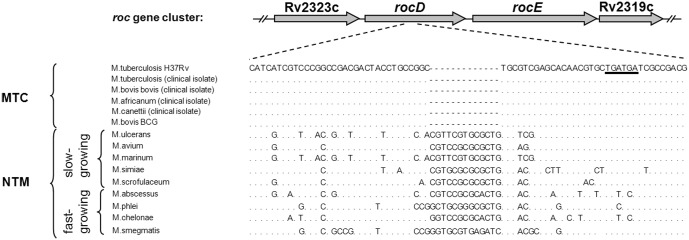
Alignment of partial *rocD* sequence from members of Mtb complex (MTC) and non-tuberculous mycobacteria (NTM). Besides published genome data for *M*. *tuberculosis* H37Rv, *M*. *ulcerans*, *M*. *avium*, *M*. *marinum*, and *M*. *smegmatis*, sequence data obtained from clinical isolates (*M*. *tuberculosis*, *M*. *bovis bovis*, *M*. *africanum*, and *M*. *canettii*) and from *M*. *bovis* BCG, *M*. *simiae*, *M*. *scrofulaceum*, *M*. *abscessus*, *M*. *phlei*, and *M*. *chelonae* were included. The genome sequence of Mtb H37Rv was used as a reference. The partial *rocD* sequence encompasses base pairs 615 to 681. Members of the Mtb complex (MTC), in contrast to non-tuberculous mycobacteria (NTM), carry a 13 bps deletion within *rocD* resulting in a premature stop (underlined).

**Fig 4:**
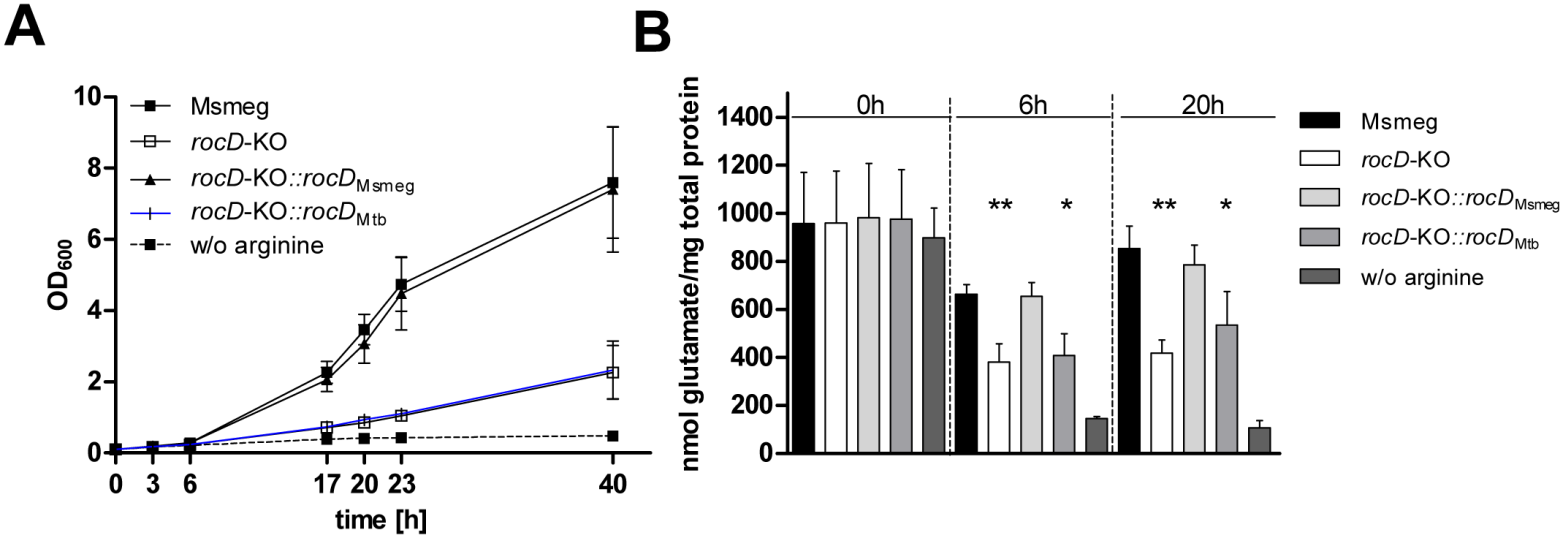
Growth (A) and glutamate formation (B) of a *rocD* mutant in Msmeg cultured on arginine as nitrogen source. Msmeg wild type (Msmeg), Msmeg knockout mutant for *rocD* (*rocD*-KO), the Msmeg *rocD*-KO mutant complemented with *rocD* wild type gene from Msmeg (*rocD*-KO::*rocD*_Msmeg_), and the Msmeg *rocD*-KO mutant complemented with *rocD* wild type gene from Mtb (*rocD*-KO::*rocD*_Mtb_) are shown. All strains were cultured for 40 hours in minimal medium with glycerol and glucose as carbon sources and with 5 mM of arginine as a sole nitrogen source (solid line). Msmeg wild type was also cultured without arginine as a control (broken line). **A)** Growth was analyzed measuring absorbance (OD_600_) at 3h, 6h, 17h, 20h, 23h, and 40h. Mean values and standard deviations are shown for three independent biological experiments. **B)** Intracellular glutamate was determined in bacterial cell extracts after 0, 6, and 20 hours of growth. Mean values and standard deviations are shown for three independent biological experiments, p- values were calculated using student’s *t* test (* p < 0.05, ** p < 0.01).

We compared the *rocD* sequence of the H37Rv laboratory strain with various clinical isolates of the Mtb complex (MTC) and other mycobacterial species. The *rocD* alignment in Fig 3 shows that members of the MTC (Mtb, *Mycobacterium bovis*, *Mycobacterium canettii*, *Mycobacterium africanum*) carried a 13 bps deletion within the *rocD* gene, resulting in a frameshift mutation. This mutation introduced two stop codons, downstream to the deletion. Interestingly, non-tuberculous mycobacteria (NTM), both the fast- and slow-growers, possessed a full-length *rocD* (Fig 3). To investigate the role of *rocD* for arginine utilization in non-tuberculous mycobacteria, we generated a *rocD*-KO mutant in *Mycobacterium smegmatis* (Msmeg). As expected, growth of the *rocD*-KO mutant in Msmeg was impaired on arginine (Fig 4A). The *rocD*-KO mutant showed less intracellular glutamate compared to the wild type (Fig 4B). Growth and glutamate levels returned to wild type levels in the *rocD*-KO mutant when genetically complemented with *rocD* of Msmeg but remained unchanged with *rocD* of Mtb (Fig 4B). Thus, lack of *rocD* in Msmeg reduces nitrogen incorporation from arginine into glutamate and compromises intracellular nitrogen levels as glutamate is the main nitrogen provider in the metabolism. These results suggest that in contrast to Mtb, the ornithine aminotransferase (*rocD*) in Msmeg mediates utilization of arginine as a nitrogen source. It also confirmed our hypothesis that the partial deletion in *rocD* of Mtb disrupts its ornithine aminotransferase function.

We had shown that *rocD* is non-functional in Mtb, the *adi*-KO mutant was not impaired in growth on arginine, but the Rv2323c-KO mutant was drastically restricted with respect to utilization of arginine. Thus, we now focused on Rv2323c and sought to compare the metabolic profile of Mtb wild type with that of the Rv2323c-KO mutant during growth on arginine.

### Mtb utilizes arginine as a carbon source to produce ornithine and proline

**Fig 5:**
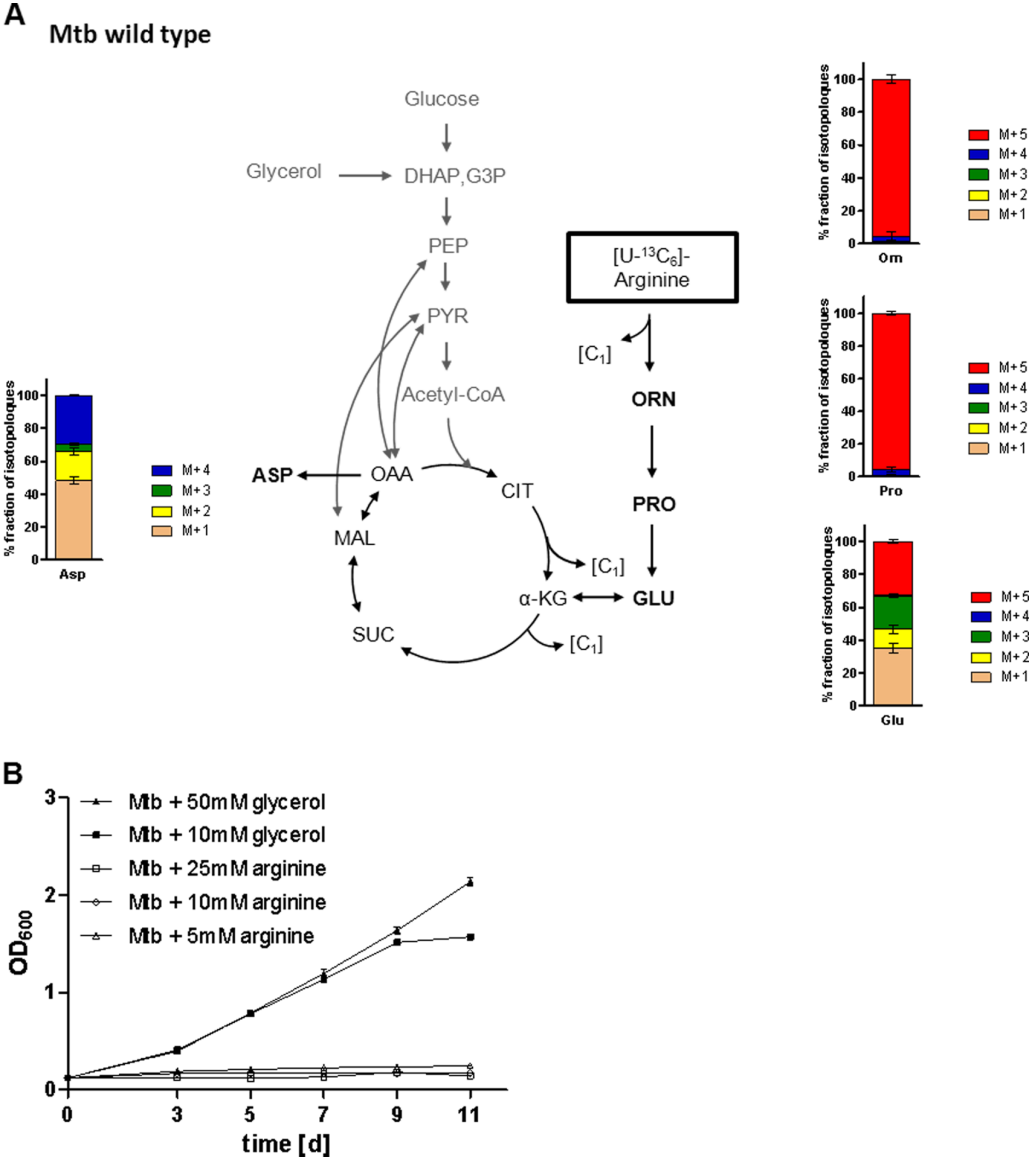
Relative isotopologue composition of amino acids derived from Mtb grown in presence of [U-^13^C_6_]arginine (A) and cultivation of Mtb on arginine as carbon source (B). **A)** Mtb wild type was cultured for 7 days in minimal medium with glycerol and glucose as carbon sources and with 5 mM of a 1:1 mixture of [U-^13^C_6_]arginine) and unlabeled arginine as sole nitrogen source. ^13^C-Enrichments of ornithine, proline, glutamate, and aspartate were determined by GC-MS. Labeled ornithine and proline contained mainly M+5 isotopomers. Glutamate and aspartate showed more complex mixtures of multiple isotopomers. M+1, M+2, M+3, M+4, and M+5 indicate molecules with one to five ^13^C- labeled atoms, respectively (for ^13^C-excess of labeled amino acids see S2 Table). Mean values and standard deviations are shown for two independent biological experiments each measured in triplicate. **B)** Mtb wild type (Mtb) was cultured for 11 days in Sauton’s modified medium with 5 mM, 10 mM, or 25 mM of arginine (open symbols), or with 10 mM or 50 mM of glycerol (closed symbols) as single carbon source. Mean values and standard deviations are shown for three independent biological experiments.

For metabolic pathway analysis, bacteria growing in minimal medium with glucose and glycerol as carbon sources were supplemented with a mixture (1:1) of [U-^13^C_6_]arginine and unlabeled arginine as sole nitrogen source. After 0, 3, 5, and 7 days of growth with the labeled substrate, the bacteria were harvested and hydrolyzed under acidic conditions. ^13^C-Enrichments of the resulting amino acids were determined by GC-MS (S2 Table). After 7 days of growth, we observed a substantial ^13^C-excess in both amino acids ornithine and proline (47.8% and 49.8% ^13^C-excess, respectively) showing that both compounds originated almost completely from the exogenously provided arginine (Fig 5A). In contrast, ^13^C-excess in glutamate exhibited only 6.2% suggesting that the carbon atoms of glutamate were predominantly provided by glucose and/or glycerol present in unlabeled form in the medium. Minor ^13^C-incorporation (1–4%) was also found in TCA cycle-derived amino acids such as aspartate, threonine, isoleucine, and lysine demonstrating that carbons from arginine can be incorporated into the central carbon metabolism. However, the ^13^C-excess values of glycolytic amino acids (alanine, glycine, leucine, phenylalanine, serine, tyrosine, valine) were below the detection level (<1%) (S2 Table).

The isotopologue composition of ornithine and proline could be inferred from the mass distributions in the MS spectra (Fig 5A). Major mass peaks were found for the M+5 fractions in both ornithine and proline (approximately 95%) showing that both compounds were predominantly labeled at all of the five C-atoms. This provided clear evidence that the synthesis of ornithine and proline proceeds from arginine by cleavage of one carbon atom. In contrast, the isotopologue profile of glutamate illustrated the presence of an isotopologue mixture mainly of those carrying one, three, or five ^13^C-atoms, respectively (M+1 [35%], M+2 [12%], M+3 [20%], M+4 [1%], M+5 [32%]). This observation supports the idea that the biosynthesis of glutamate is based on α-ketoglutarate directly derived from arginine (i.e. giving rise to the M+5 isotopomer), but also from the TCA cycle involving selectively labeled precursors (i.e. giving rise to the M+1 and M+3 isotopomers). Similar to glutamate, the multiple isotopologue profile of aspartate shows its synthesis from arginine on the one hand and from the principle supplemented carbon sources glucose / glycerol on the other. However, arginine did not promote cell growth of Mtb when added as sole carbon source in a defined minimal medium (Fig 5B).

Mtb uses carbons from arginine to produce ornithine, proline, and other amino acids such as glutamate and aspartate. To examine the function of Rv2323c in the arginine catabolism, we next analyzed the production of these amino acids in the Rv2323c-KO mutant when growing on arginine.

### Rv2323c is involved in arginine utilization

**Fig 6:**
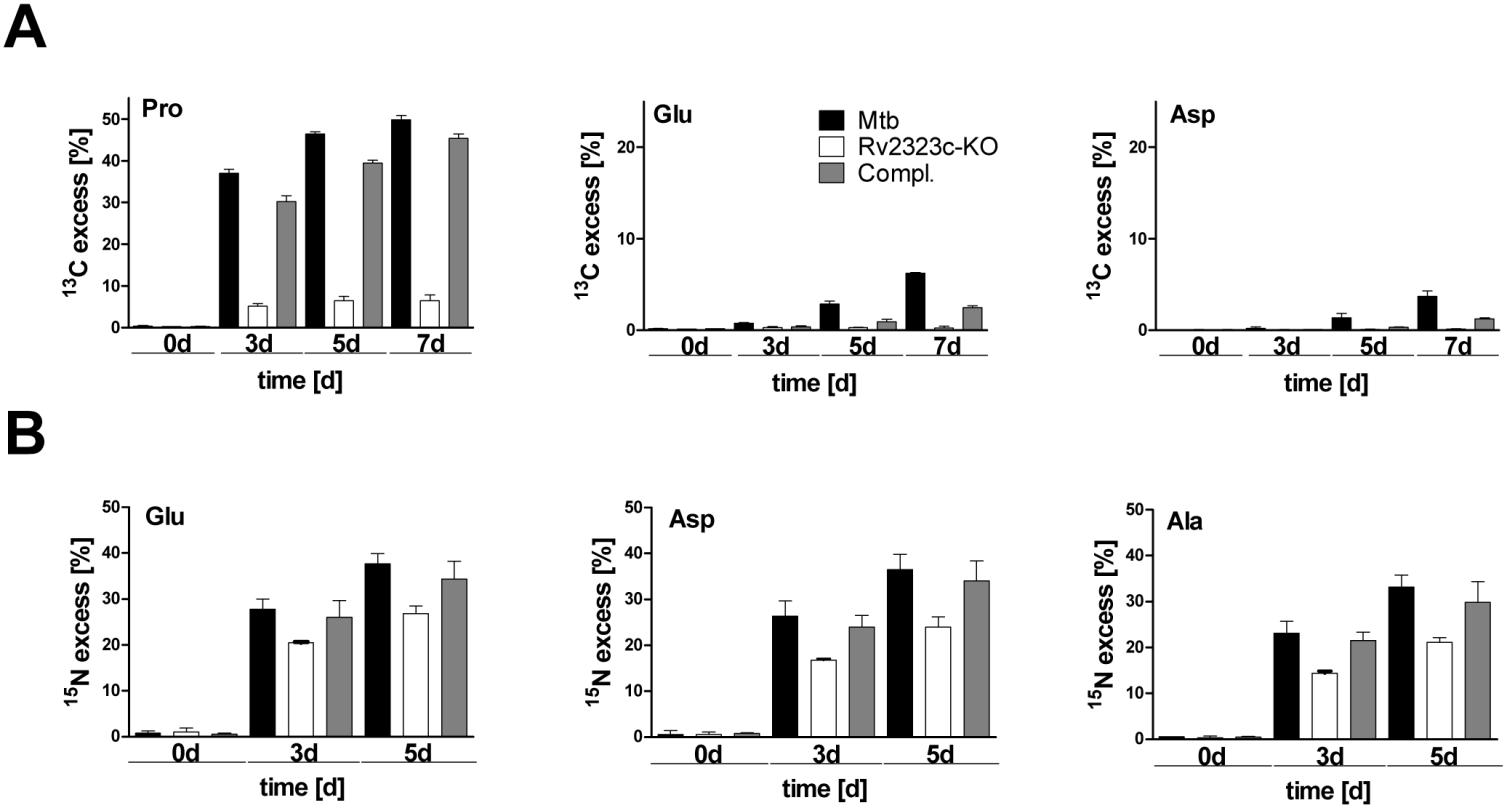
^13^C- or ^15^N- excess of labeled amino acids in a Rv2323c knockout strain grown with [U-^13^C_6_]arginine (A) or [U-^15^N_4_]arginine (B). Mtb wild type (Mtb), Rv2323c knockout mutant (Rv2323c-KO), and the Rv2323c knockout mutant complemented with the Rv2323c wild type gene (Compl.) are shown. Bacteria were cultured in minimal medium with glucose and glycerol as carbon sources and with either 5 mM of a 1:1 mixture of [U-^13^C_6_]arginine and unlabeled arginine as sole nitrogen source (**A)** or with 5 mM of a 1:1 mixture of [U-^15^N_4_]arginine and unlabeled arginine as sole nitrogen source **(B). A)** The ^13^C-excess of proline and other selected amino acids such as glutamate and aspartate is shown after 0, 3, 5, and 7 days of growth.^13^C-Excess at day 0 was <1%. **B)** The ^15^N-excess of glutamate and other selected amino acids such as aspartate and alanine is shown after 0, 3, and 5 days of growth (for more details see S3 Table). ^15^N-Excess at day 0 was < 1%. Mean values and standard deviations are shown for two independent biological experiments each measured in triplicate.

To elucidate the role of Rv2323c in carbon utilization from arginine, we cultured Mtb wild type and the Rv2323c-KO mutant in minimal medium containing glucose and glycerol supplemented with a mixture (1:1) of [U-^13^C_6_]arginine and unlabeled arginine as sole nitrogen source. The Rv2323c-KO mutant exhibited a greatly reduced carbon flow from arginine into proline, glutamate, and aspartate (Fig 6A). After 7 days of growth, the overall ^13^C-excess in proline was only 6.4% for the Rv2323c-KO mutant whereas the wild type showed 49.8%. Furthermore, carbon incorporation from arginine into glutamate and aspartate exhibited 6.2% and 3.7% for the wild type whereas ^13^C-excess in both amino acids was abolished in the Rv2323c-KO mutant strain (<1%). This clearly indicates an essential role of Rv2323c in the arginine catabolism of Mtb.

To investigate the role of Rv2323c in nitrogen utilization from arginine, we provided a mixture (1:1) of [U-^15^N_4_] arginine and unlabeled arginine to growing Mtb in minimal medium. We observed a uniform ^15^N-excess value in all amino acids of approximately 40% for the wild type (S3 Table). For example, as depicted in Fig 6B, the ^15^N-enrichment in glutamate was 37.7% after 5 days of growth for the wild type. For aspartate, a TCA cycle derived amino acid, and for the glycolytic amino acid alanine a ^15^N-excess of 36.5% and 33.2% was determined. In comparison to Mtb wild type, the Rv2323c-KO mutant showed lower ^15^N-excess values for glutamate (26.9%), aspartate (23.9%), and alanine (21.1%) (Fig 6B), and also for all the other amino acids that were measured (S3 Table). This again confirms that Rv2323c metabolizes arginine, and in addition to carbons provides nitrogen for the central nitrogen metabolism of Mtb. However, the Rv2323c-KO strain maintained a residual capability to incorporate nitrogen (^15^N) from arginine indicating that Mtb possesses an alternative pathway to degrade arginine as nitrogen source.

## Discussion

It is known that arginine serves as a nitrogen source for growth of Mtb *in vitro* but the pathways and molecular mechanisms for arginine utilization were unsolved. We now show that in the presence of arginine, Mtb induces a gene cluster, which includes homologues for an arginine transporter (*rocE*), an ornithine aminotransferase (*rocD*) and two genes of unknown function, Rv2323c and Rv2319c. Whereas *rocD* carries a deletion causing a frameshift mutation, Rv2323c turned out to be crucial for growth of Mtb on arginine. Following the relative fluxes of ^15^N- or ^13^C-arginine, we observed that arginine serves as a universal nitrogen source, and also as a carbon source for certain amino acids such as proline, and that these pathways depend on Rv2323c.

With respect to the regulation of genes involved in arginine metabolism, Gardan and coworkers showed that the arginine-dependent induction of the *rocDEF* operon in *B*. *subtilis* is controlled by the transcriptional regulator *rocR* [26]. *RocR* is located next to *rocDEF* and is transcribed into opposite direction. Similar to the genome organization of the *roc* region in *B*. *subtili*s, in Mtb Rv2324, which is annotated as a transcriptional regulator, follows immediately the Rv2319c-*rocDE*-Rv2323c gene cluster [22]. In addition, we found that Rv2324 is 6-fold upregulated in presence of arginine (S1 Table), also pointing to a role of Rv2324 in arginine metabolism of Mtb.

The *nirBD* operon, which encodes the nitrite reductase and is involved in nitrate and nitrite assimilation of Mtb, was also induced in presence of arginine. We have shown previously that *nirBD* expression in Mtb is positively regulated by *glnR* [29], which is described as a global regulator of nitrogen metabolism in other bacteria [32]. Jenkins and colleagues showed that *rocDE* is not part of the *glnR* regulon in Msmeg [33]. A different study demonstrated that a *glnR* mutant in Msmeg has no growth defect on arginine [34]. Nonetheless, a link between arginine and nitrite metabolism might be provided by recent observations that inside human macrophages, Mtb utilizes nitrate originating from the arginine-metabolism (iNOS) of the host to form nitrite [11]. Therefore, when Mtb encounters arginine, it might be appropriate to activate both arginine catabolic enzymes such as Rv2319c-*rocDE*-Rv2323c as well as *nirBD* for metabolizing nitrite.

On the basis of isotopologue profiling experiments, arginine can be efficiently converted into ornithine by Mtb growing in defined minimal medium. However, the genome of Mtb does not include a gene with homology to an arginase enzyme, which in many other bacteria produces ornithine from arginine via hydrolysis. As the function of at least 25% of the coding sequences in Mtb has yet to be determined [31], we cannot exclude that Mtb contains an arginase. Alternatively, Mtb might use another mechanism for ornithine formation from arginine, such as arginine amidinotransferases [35–37].

The isotopologue profiling analysis also demonstrated that arginine served as efficient source for the production of proline in Mtb. In *Staphylococcus aureus* it has been shown that proline is produced from arginine via ornithine, rather than from glutamate [38]. A proline auxotrophic strain of *B*. *subtilis* assembled mutations in the *rocDEF* region, and thereby increased the conversion of arginine via the arginase pathway. This maintained the intracellular proline pool and restored growth of the proline auxotrophic mutant to wild type level [27]. Proline is typically synthesized from arginine via the arginase pathway [24,26,38]. As shown in this study, the ornithine aminotransferase (*rocD*), which is essential for the arginase pathway, is naturally deleted in Mtb. Thus, Mtb must have an alternative route to convert arginine into proline. The genome of Mtb includes further homologues for aminotransferase enzymes (Rv0075, Rv0812, Rv1178, Rv2294, Rv3329, and Rv3778) with unknown substrate specificity that might compensate for the loss of *rocD* [22]. However, none of them has been found to be upregulated in presence of arginine (S1 Table).

Rv2323c comprises 909 base-pairs and has been annotated as a dimethylarginine dimethylaminohydrolase. This enzymatic activity has been extensively studied in eukaryotic cells, and cleaves methylated arginine into dimethylamine and citrulline. Rv2323c shows homology to dimethylarginine dimethylaminohydrolase from *Streptomyces coelicolor* SC5F2A.01c [22]. To the best of our knowledge, there are no functional studies of dimethylarginine dimethylaminohydrolases in prokaryotes. Besides the role of Rv2323c for utilization of arginine as a carbon source for individual amino acids, it is also involved in providing nitrogen from arginine, as in the Rv2323c-KO mutant all amino acids that we measured showed a reduced rate of ^15^N-labeling. The growth defect of the Rv2323c-KO mutant on arginine is therefore most likely due to its restriction in nitrogen assimilation under arginine conditions.

In conclusion, this study contributes to the understanding of the arginine metabolism in Mtb. Both nitrogen and carbons from arginine can be incorporated into the central metabolism of Mtb. Arginine serves as a general nitrogen source, and also as a carbon source for selected amino acids such as proline. This study also introduces Rv2323c to the arginine catabolism of Mtb, and showed that a frameshift mutation in *rocD* turned out to be unique for members of the Mtb complex. In contrast to other mycobacteria, members of the Mtb complex are typically restricted to mammals, in particular to humans, and lack an environmental life style. Further studies will show whether these features reflect the difference in the various life styles of mycobacteria and whether Rv2323c represents a new metabolic target for tuberculostatics.

## Materials and Methods

### Bacterial Strains and Media

Mtb H37Rv and Msmeg mc^2^ 155 were cultured in 7H9 broth or on 7H10 plates (Difco Laboratories), supplemented with 0.5% glycerol and 10% ADS (50 g/L Bovine Albumin Fraction V, 20 g/L D-glucose, 8.1 g/L NaCl). To test growth on single nitrogen sources, bacteria were grown in minimal medium (10% basic salts, 0.2% trace elements, 0.5 mM MgCl_2_, 0.5 mM CaCl_2_), supplemented with 0.5% glycerol, 10% ADS, and 5 mM of the indicated nitrogen source. Basic salts combined 10 g/L KH_2_PO_4_, 25 g/L Na_2_HPO_4_ and 20 g/L K_2_SO_4_. The trace elements solution contained 40 mg/L of ZnCl_2_, 200 mg/L of FeCl_3_, 10 mg/L of CuCl_2_, 10 mg/L of MnCl_2_, and 10 mg/L of Na_2_B_4_O_7_. Growth in presence of single carbon sources was performed in Sauton’s modified medium [39] supplemented with the indicated concentrations of arginine or glycerol. For all liquid media, 0.05% tyloxapol was added. Mycobacteria were incubated in roller bottles (Corning) at 70 rpm (Innova 4000 Benchtop Incubator Shaker, New Brunswick Scientific).

### Transcriptome Analysis

Mtb was aerobically cultured in minimal medium supplemented with 5 mM of arginine or 5 mM of ammonium chloride as sole nitrogen sources to an optical density of OD_600_ = 0.8 using roller bottles. Fold change in gene expression during growth on arginine was calculated by comparing gene expression between Mtb grown on either nitrogen source (ammonium chloride was used as a reference). Total RNA was prepared from 60 mL of bacterial culture incubated in an equal volume of GTC buffer (5.1 M guanidine thiocyanate, 17 mM n-lauroylsarcosine, 28.3 mM sodium citrate, 0.7% ß-mercaptoethanol) for 15 min at room temperature. Cell pellets were resuspended in trizol reagent, disrupted mechanically by beat beating, and subjected to centrifugation (16,000 × g, 10 min). RNA was isolated using the RNeasy Kit from Qiagen including the optional on-column DNaseI digestion for 1 h. Remaining DNA was removed by an additional off-column DNaseI (NEB) digestion for 45 min. RNA integrity was determined using an Agilent Bioanalyzer 2100.

10 μg of RNA was transcribed into cDNA using the SuperScript II reverse transcriptase with random hexamer primers (Invitrogen), fragmented (DNaseI from Thermo Scientific), and labeled using the terminal deoxynucleotidyl transferase (Promega) together with the GeneChip DNA Labeling Reagent from Affymetrix. cDNA was purified using the QIAquick PCR Purification kit (Qiagen). cDNA was hybridized for 16 h at 50°C on a custom-designed microarray slide (GeneChip.MTbH37Rva520456F), stained (Affymetrix Fluidics Station 450 Flex, FS450_0002 Pae_G1a protocol), and scanned using the Gene Chip Scanner 3000. Signal intensities were quantified using the GCOS 3.1 software (Affymetrix) with standard settings. Probeset summarization and microarray normalization was performed by RMA algorithm. Data have been submitted to NCBI’s Gene Expression Omnibus and are accessible via the GEO Series accession number GSE67445 (http://www.ncbi.nlm.nih.gov/geo/query/acc.cgi?acc=GSE67445). Data of three biological replicates of each cultivation condition (including also one set of technical replicates, which were averaged) were grouped and compared, using the *limma* R package tool. [40]. Multiple-testing corrections on p-values were included using the Benjamini & Hochberg false discovery rate method. An absolute fold change > 2 and a corrected p-value cut-off of < 0.05 were used to determine significant differential gene expression.

### Sequence Analysis of *RocD*

For sequence analysis of *rocD*, we used published sequences of Mtb H37Rv, *Mycobacterium ulcerans* Agy 99, *Mycobacterium avium* 104, *Mycobacterium marinum* M, and Msmeg mc² 155 (http://genolist.pasteur.fr/GenoList). In addition, we amplified and sequenced the *rocD* gene of *Mycobacterium abscessus* ATCC 19977, *Mycobacterium phlei* ATCC 11758, *Mycobacterium chelonae* ATCC 35753, *Mycobacterium scrofulaceum* ATCC 19961, and *Mycobacterium simiae* ATCC 19420 using degenerate oligonucleotides: 479 (5’-ccg yaa rtg ggg gvr ccg asg tc-3’) and 481 (5’-ttk ccr ccg aab gts gas ccg-3’), that we designed according to published sequences. Oligonucleotides that target the published sequence of Mtb H37Rv were used for clinical isolates of Mtb, *Mycobacterium bovis*, *Mycobacterium africanum*, and *Mycobacterium canettii*, as well as *Mycobacterium bovis* BCG Pasteur: 530 (5’-ccgcaagtggggagccgacgtc-3’) and 531 (5’-ttgccgccgaacgtcgacccg-3’). Primer 479 and 530 were used for sequencing.

### Generation of Knockout Strains

The used method is described in Pavelka *et al*. [41]. Cosmids, carrying *rocD*, *adi*, or Rv2323c, were obtained from a genomic library of Mtb H37Rv or Msmeg mc² 155 [42] by colony blot hybridization. The gene of interest was subcloned in vector pMV306.kan or pBSK(-), a 1723 bps deletion in *rocD* of Mtb or a 940 bps deletion in *rocD* of Msmeg was generated by enzyme digestion. A 2009 bps fragment and a 484 bps fragment were deleted in *adi* and Rv2323c. The knockout region was cloned in the suicide plasmid pMP62, equal to pYUB657 in Pavelka *et al*., which was used for transformation of Mtb or Msmeg. 7H10 plates supplemented with 4% sucrose were used to obtain clones, in which the two recombination events had occurred. Mutants were confirmed by Southern blot hybridization. For complementation, the knockout strains were transformed with *rocD* or Rv2323c, including the native promoter, cloned in pMV306.hyg.

### Labeling Experiments

Mtb was cultured in minimal medium containing 1:1 mixtures of 5 mM unlabeled and completely labeled ^13^C_6_- or ^15^N_4_-arginine, respectively (Silantes). At indicated time points, approximately 10^9^ cells were harvested and the cell pellet was washed with 1x PBS and autoclaved. Protein hydrolysis and amino acid derivatization: The lyophilized bacterial cell pellet (2 mg, dry weight) was suspended in 0.5 mL of 6 M HCl and heated at 105°C for 24 h under inert atmosphere. The hydrolysate was placed onto a cation exchange column of Dowex 50W × 8 (H^+^ form, 200–400 mesh, 5 x 10 mm), which was washed with 1 mL of 70% methanol, afterwards with 2 mL of water, and subsequently developed with 1 mL of 4 M ammonium hydroxide. A 0.5 mL aliquot of the eluate was dried under a nitrogen stream and then dissolved in 50 μl of water-free acetonitrile. An equal volume of *N*-(tert-butyldimethyl-silyl)-*N*-methyl-trifluoroacetamide containing 1% tert-butyl-dimethyl-silylchloride was added and the mixture was incubated at 70°C for 30 min. The resulting *N*-(tert-butyldimethylsilyl) (TBDMS) derivatives of amino acids were then analyzed by GC/MS. GC/MS analysis: GC/MS was performed on a GCMS-QP2010 Plus Gas Chromatograph/Mass Spectrometer (Shimadzu) equipped with a fused silica capillary column (Equity TM-5, 30 m by 0.25 mm, 0.25-μm film thickness, Supelco) working with electron impact ionization at 70 eV. One μL of a TBDMS derivative solution was injected in a 1:10 split mode at an interface temperature of 260°C and a helium inlet pressure of 70 kPa. The column was then developed at 150°C for 3 min with a temperature gradient of 7°C/min to a final temperature of 280°C that was held for 3 min. Data were collected using the LabSolutions software (Shimadzu). Selected ion monitoring data were acquired according to Eylert *et al*. using a 0.3-s sampling rate [43].

Ornithine was analyzed as ornithine-TFA-methylester by GC/MS analysis [44]. For this purpose, another 0.5 mL aliquot of the cation exchange eluate (see above) was dried under a stream of nitrogen and dissolved in 200 μL of methanolic 3 N HCl. The mixture was heated to 70°C for 30 min and dried under a stream of nitrogen afterwards. The residue was dissolved in 50 μL of trifluoroacetic acid and heated to 140°C for 10 min. The mixture was dried again, dissolved in 100 μL of anhydrous ethylacetate, and subjected to GC/MS analysis. General GC/MS conditions were the same as described for TBDMS amino acid derivatives (see above). For TFA-methylester derivatives, the column was held at 70°C for 3 min and then developed with a temperature gradient of 10°C min^-1^ to a final temperature of 200°C that was held for 3 min. The retention time for the ornithine-TFA-methylester was R_t_ = 14.0 min. The observed fragment for ^13^C-excess calculations for ornithine was m/z 306 corresponding to [M-CH_3_OH]^+^. The overall ^13^C-excess (mol%) and the relative abundances of isotopomers were computed by an Excel-based in-house software package according to Lee *et al*. [45].

### Quantification of Glutamate

20 mL of bacterial culture were harvested by centrifugation. Cell pellets were washed in 1x PBS, resuspended in 600 μL *Aq*.*dest*. and heated for 30 min at 90°C. Cells were mechanically disrupted by beat-beating. Cell lysates were cleared by centrifugation using centrifugal filter units (0.22 μm, Millipore). Glutamate was determined by an enzymatic test from Boehringer Mannheim/R-Biopharm according to the protocol of the manufacturer.

### Software and Statistics

DNA-Star lasergene 9.0 was used for alignments. The student’s *t*-test was used for statistical analyses. P-values < 0.05 were considered to be significant.

## Supporting Information

S1 Fig Growth of the Rv2323c-KO mutant on glutamate. Mtb wild type (Mtb), closed squares, and the Rv2323c knockout mutant (Rv2323c-KO), open squares, are shown. Bacteria were cultured in minimal medium with 5 mM of glutamate as sole nitrogen source (solid line) or without glutamate (broken line). Mean values and standard deviations are shown for three independent biological experiments.
(TIF)

S1 Table Whole genome expression profiling of Mtb grown in the presence of arginine. Bacteria were cultured in minimal medium with glucose and glycerol as carbon sources and with either arginine or ammonium chloride as sole nitrogen source to an optical density of OD_600_ = 0.8. RNA was prepared, transcribed into cDNA, labelled, and subjected to microarray analysis. Fold change (FC) was calculated by comparing the signal intensities obtained from bacteria cultured in the presence of arginine with bacteria cultured in the presence of ammonium chloride. The latter was used as a reference. Depicted are all genes with an absolute fold change (absFC) > 2 and a corrected p-value (adj.P.Val) < 0.05.
(XLSX)

S2 Table ^13^C-Excess values of labelled amino acids during growth of Mtb with [U-^13^C_6_]arginine. The ^13^C-excess values correspond to the experiment shown in Fig 5A. ^13^C-Incorporation by Mtb wild type (Mtb) from arginine into amino acids was determined after 0, 3, 5, and 7 days of growth. Mean values and standard deviations are shown for two independent biological experiments each measured in triplicate.
(XLSX)

S3 Table ^15^N-Excess data of labelled amino acids during growth of Mtb with [U-^15^N_4_]arginine. The ^15^N-excess values correspond to the experiment shown in Fig 6B. Mtb wild type (Mtb), Rv2323c knockout mutant (Rv2323c-KO), and the Rv2323c knockout mutant complemented with the Rv2323c wild type gene (Compl.) are shown. ^15^N-Incorporation by bacterial strains from arginine into amino acids was determined after 0, 3, and 5 days of growth. Mean values and standard deviations are shown for two independent biological experiments each measured in triplicate.
(XLSX)
