# Supplementary figures and images for: Mycobacterium tuberculosis Is a Natural Ornithine Aminotransferase (rocD) Mutant and Depends on Rv2323c for Growth on Arginine
Source: PLoS One. 2015 Sep 14;10(9):e0136914. doi: 10.1371/journal.pone.0136914 (PMC4569260; doi:10.1371/journal.pone.0136914)

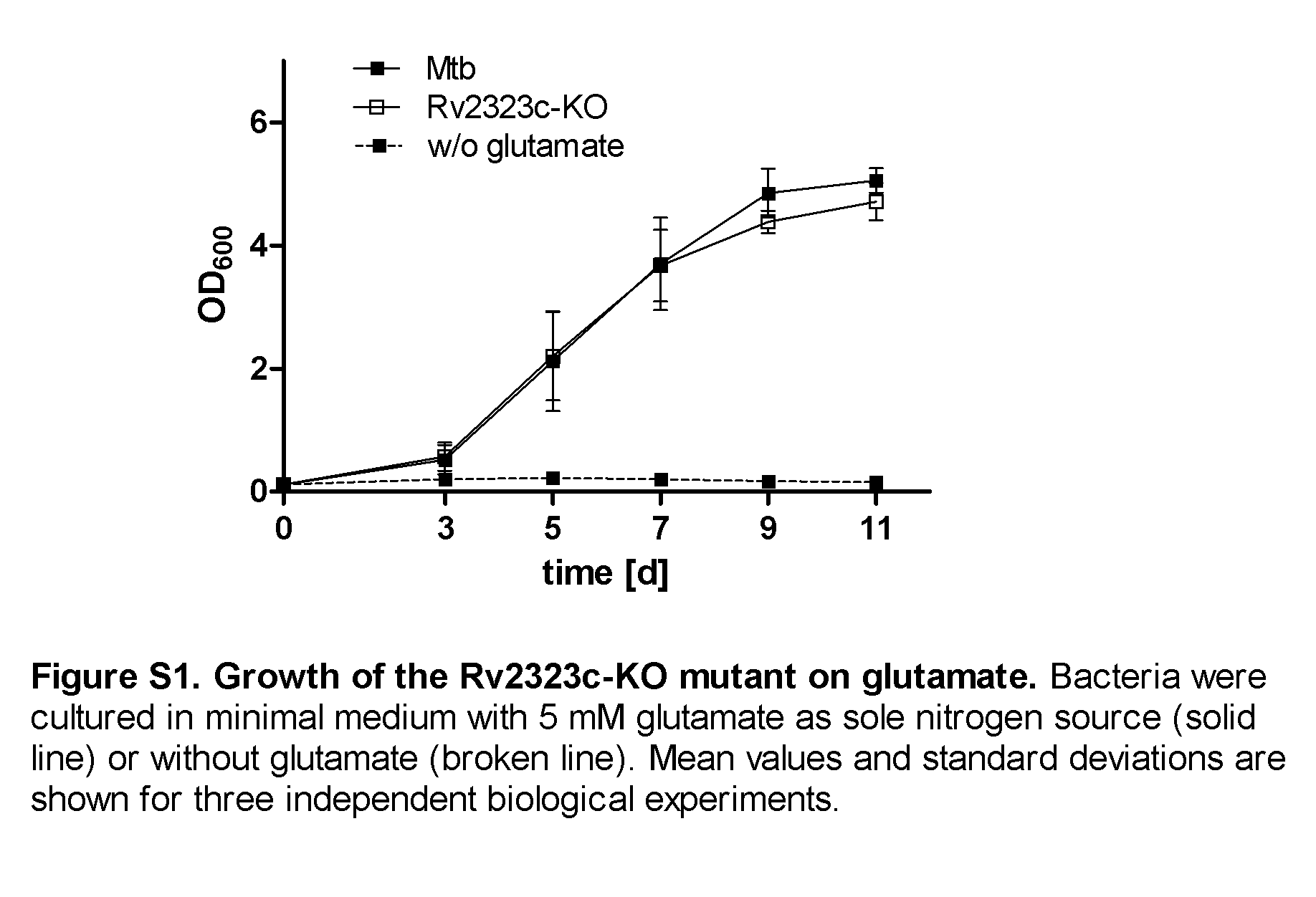

Supplement: S1 Fig — Mtb wild type (Mtb), closed squares, and the Rv2323c knockout mutant (Rv2323c-KO), open squares, are shown. Bacteria were cultured in minimal medium with 5 mM of glutamate as sole nitrogen source (solid line) or without glutamate (broken line). Mean values and standard deviations are shown for three independent biological experiments. (TIF) [file pone.0136914.s001.tif]
